# Hospital Co-Operation and Mutual Help

**Published:** 1886-11-27

**Authors:** Andrew Clark


### Hospital Co-operation and Mutual Help.

By Sir Andrew Clark, Bart., M.D., F.R.S.

A Cordial Welcome by the Association.

At the Hospitals Association Conversazione on Wednesday last, Sir Andrew Clark said that he had been commanded by the Council of the Hospitals Association to undertake two very agreeable duties. The first was to offer to all assembled a most cordial welcome, and to convey to them the grateful thanks of the Council for the honour conferred upon the Association by their presence there that evening. There were present representatives, first of the great hospitals of this city, next of the smaller hospitals elsewhere, thirdly of the asylums, and lastly of the nursing institutions. In the name of the Council of the Hospitals Association, he again bade them all and each a cordial welcome. Especially would he thank the ladies whom he saw before him, not only because their presence was as sunshine in the congregation of men, but for another and a higher reason. Women could give to the work in which they were engaged something which men could not bestow. Mere intellect and mere logic were not the sole factors in the elucidation of truth, or the only guides in the practical conduct of life. Men, in the management of affairs, were for the most part guided by understanding and experience, but women, to a large extent, were guided by instinct and feeling. Both were essential in the conduct of human affairs, and hence it was in the combination of the two that we had the largest promise of the highest good in those works where these orders were found together. Therefore it was that he accorded an especially cordial greeting to the ladies present.

For the Ladies a Special Welcome.

All the world is interested in Hospitals.

He supposed there was no one who pretended to the smallest concern in the misfortunes and sufferings of others who did not take some sort of interest in the work and life of the great hospitals of this country. Without magnifying that work, he might say of it that it was truly great. Next only to that which concerned itself with the spiritual life of man, it stood forth the highest of all possible works. For he supposed there was no work which affected human interests more widely and more deeply than this. Nay, there was hardly one human being, either old or young, rich or poor, who did not directly, or indirectly, come within the influence of hospital work, whether that influence were for good or for evil.

The Special Objects of Hospitals.

Sir Andrew next dwelt upon the various objects to be considered in connection with hospital work. Chief of all should, of course, be the endeavour to relieve the sick of their suffering. Next came the enlarging and improving of medical knowledge, and thirdly the training and discipline of the medical men who were to take the place of those now at work. There was yet another matter to be borne in mind, and that was the training of nurses. Thirty years ago this subject was never dreamt of. He believed that the hospitals of this country, on the whole, did their work well, but they had their imperfections, and one of these imperfections was due to their isolation one from the other.

The Isolation of Hospitals.

Each hospital had its own individuality. But though he would not efface this individuality, he believed that their very autonomy, their strong individuality, their isolated republicanism—had its disadvantages. To those who founded the institution which he had the honour to represent, it seemed very desirable that the hospitals should come together, and that each should seek from the other what that other had to give.

The Need and Value of Co-operation.

Such intercommunication would benefit them in many ways. One hospital would ask how did you proceed in this critical period, and another how in that; they would compare notes and so forth. Each would thus be led to strive to make its management and work more efficient and the cost of its administration smaller. The Hospitals Association was founded with the great object of bringing hospitals together. It had passed through a most successful infancy and advanced to a vigorous childhood, but it was now entering upon another peril—the peril of incipient manhood. Although they had done well up to that time, they were anxious to avoid the perils before them, and those perils could only be avoided primarily by the continued wisdom of the Council and the energy of Mr. Burdett, and then also by their friends continuing to help and, through their influence, obtaining the support of those at present outside. He believed that with such co-operation they might make hospital work both more efficient and less costly, and might enable the poor to get at the sort of relief they wanted with greater promptitude. The success of the Association had begot the JOURNAL. The institution was now represented by a weekly newspaper which, like its parent, had achieved a remarkable success. Among its contributors had been the Duke of Cambridge, who, he believed, had written his maiden article in THE HOSPITAL. Then there was Lord Leigh; and he heard that they were going to have something in it by no less a person than the Prime Minister, if, indeed, they had not had it already. They wished to be guided by the genuine enthusiasm of all those who had at heart the great work they had undertaken. Among those who desired to take a real part in the work, there must be no self-seeking, but each in his measure and her measure must give honest and diligent service to one of the noblest works which a man or an association could take in hand. He commended to them the great ideas of co-operation and mutual help for the promotion and furtherance of which the Hospitals Association had been founded and was to be maintained.

The New Journal, "The Hospital."

